# Análise de Escores de Risco para Predição de Mortalidade em Pacientes Submetidos à Cirurgia Cardíaca por Endocardite

**DOI:** 10.36660/abc.20190050

**Published:** 2020-04-06

**Authors:** Fernando Pivatto, Clarissa Carmona de Azevedo Bellagamba, Eduardo Gatti Pianca, Fernando Schmidt Fernandes, Maurício Butzke, Stefano Boemler Busato, Miguel Gus

**Affiliations:** 1 Hospital de Clínicas de Porto AlegrePorto AlegreRSBrasilHospital de Clínicas de Porto Alegre, Porto Alegre, RS – Brasil

**Keywords:** Procedimentos Cirúrgicos Cardiovasculares/mortalidade, Endocardite/complicações, Mortalidade Hospitalar, Medição de Risco

## Abstract

**Fundamento:**

Escores de risco estão disponíveis para uso na prática clínica diária, mas saber qual deles escolher é ainda incerto.

**Objetivos:**

Avaliar o EuroSCORE logístico, o EuroSCORE II e os escores específicos para endocardite infecciosa STS-IE, PALSUSE, AEPEI, EndoSCORE e RISK-E na predição de mortalidade hospitalar de pacientes submetidos à cirurgia cardíaca por endocardite ativa em um hospital terciário de ensino do sul do Brasil.

**Métodos:**

Estudo de coorte retrospectivo incluindo todos os pacientes com idade ≥ 18 anos submetidos à cirurgia cardíaca por endocardite ativa no centro do estudo entre 2007 e 2016. Foram realizadas análises de calibração (razão de mortalidade observada/esperada, O/E) e de discriminação (área sob a curva ROC, ASC), sendo a comparação das ASC realizada pelo teste de DeLong. P < 0,05 foi considerado estatisticamente significativo

**Resultados:**

Foram incluídos 107 pacientes, sendo a mortalidade hospitalar de 29,0% (IC95%: 20.4-37.6%). A melhor razão de mortalidade O/E foi obtida pelo escore PALSUSE (1,01, IC95%: 0,70-1,42), seguido pelo EuroSCORE logístico (1,3, IC95%: 0,92-1,87). O EuroSCORE logístico apresentou o maior poder discriminatório (ASC 0,77), significativamente superior ao EuroSCORE II (p = 0,03), STS-IE (p = 0,03), PALSUSE (p = 0,03), AEPEI (p = 0,03) e RISK-E (p = 0,02).

**Conclusões:**

Apesar da disponibilidade dos recentes escores específicos, o EuroSCORE logístico foi o melhor preditor de mortalidade em nossa coorte, considerando-se análise de calibração (mortalidade O/E: 1,3) e de discriminação (ASC 0,77). A validação local dos escores específicos é necessária para uma melhor avaliação do risco cirúrgico. (Arq Bras Cardiol. 2020; 114(3):518-524)

## Introdução

Apesar dos avanços no tratamento, a endocardite infecciosa (EI) está associada a morbidade e mortalidade significativas.^1^ A correção cirúrgica da EI ativa está associada à maior taxa de mortalidade dentre todas as doenças valvares, com taxas globais de mortalidade hospitalar acima de 20%.^2^

A cirurgia é atualmente realizada em 50 a 60% dos pacientes com EI.^3^ As indicações são: insuficiência cardíaca (geralmente relacionada à disfunção valvar), infecção não controlada (geralmente associada à extensão perivalvular e a defeitos de condução atrioventricular), e prevenção da embolia sistêmica.^4^ Embora essas indicações sejam claras, a sua aplicação prática baseia-se em grande parte na condição clínica, nas comorbidades, e no risco operatório do paciente.^5^

Modelos de predição de risco para cirurgia cardíaca vêm sendo desenvolvidos para fornecer informações acerca dos riscos tanto para os médicos quanto para os pacientes, bem como para guiar a tomada de decisão.^6^ A avaliação do risco cirúrgico ajuda a medir a qualidade do serviço de saúde, e o perfil de risco é essencial para diferenciar os pacientes de acordo com a gravidade da condição de saúde. Do mesmo modo, conhecer o risco do paciente pode permitir a implementação de estratégias individualizadas, visando prevenir complicações.^7^ Embora haja escores de risco disponíveis para uso na prática clínica diária, ainda há muita incerteza sobre qual deles escolher. Nesse contexto, o objetivo deste estudo foi avaliar o EuroSCORE logístico,^8^ o EuroSCORE II^9^ e escores específicos para a EI, STS-IE,^2^ PALSUSE,^10^ AEPEI,^11^ EndoSCORE^7^ e RISK-E,^12^ como preditores de mortalidade hospitalar em pacientes submetidos à cirurgia cardíaca em razão de EI ativa, em um hospital universitário terciário da região sul do Brasil.

## Métodos

Este estudo de coorte retrospectivo incluiu todos os pacientes com ≥ 18 anos de idade que foram submetidos à cirurgia cardíaca em razão de EI ativa, no Hospital de Clínicas de Porto Alegre (HCPA), de 2007 a 2016. Apenas pacientes com EI definitiva diagnosticada com base nos critérios de Duke modificados^13^foram incluídos. Os pacientes foram identificados através dos agendamentos cirúrgicos e da busca por palavras-chave no sistema eletrônico de registros médicos do HCPA. O presente estudo foi aprovado pelo Comitê de Ética em Pesquisa da instituição (protocol n^o^ 16-0632).

O risco cirúrgico pré-operatório foi calculado através da média do EuroSCORE^8^ logístico e do EuroSCORE II,^9^ além dos escores específicos STS-IE,^2^ PALSUSE,^10^ AEPEI,^11^EndoSCORE^7^ e RISK-E^12^ (Tabela 1). A mortalidade durante a internação, independente do tempo de permanência, foi definida como mortalidade hospitalar. O clearance de creatinina (CC) foi estimado através da fórmula de Cockcroft-Gault.^14^

**Tabela 1:** – Escores específicos para endocardite infecciosa analisados no presente estudo

ESCORES NÃO ESPECÍFICOS				
EuroSCORE, 1999^8^			EuroSCORE II, 2012^9^	
Endocardite ativa			Endocardite ativa	
Idade			Idade	
Estado crítico no pré-operatório			Angina CCS classe 4	
Cr sérica > 200 µmol/L			Doença pulmonar crônica	
Arteriopatia extracardíaca			Estado crítico no pré-operatório	
Sexo feminino			Arteriopatia extracardíaca	
FEVE			Sexo feminino	
Disfunção neurológica			DMID	
Cirurgia não coronariana			FEVE	
Doença pulmonar			Classe funcional (NYHA)		
Cirurgia cardíaca prévia			Mobilidade reduzida	
IAM recente			Cirurgia cardíaca prévia	
PSAP > 60 mmHg			IAM recente	
Cirurgia na aorta torácica			Disfunção renal	
Angina instável			PSAP	
Urgência			Cirurgia na aorta torácica	
Ruptura do septo ventricular			Urgência	
			Procedimento realizado	
**ESCORES ESPECÍFICOS PARA EI**				
**STS-IE, 2011**^**2**^	**PALSUSE, 2014**^**10**^	**AEPEI, 2017**^**11**^	**EndoSCORE, 2017**^**12**^	**RISK-E, 2017**^**13**^
Endocardite ativa	EI de valva protética	IMC > 27Kg/m^2^	Idade	Insuficiência renal aguda
Arritmia*	Idade	Estado crítico no pré-operatório	DPOC	Idade
Choque cardiogênico	Grande destruição intracardíaca^†^	eTFG < 50mL/min	Cr sérica ≥ 2mg/dL	Choque cardiogênico
Doença pulmonar crônica	Staphylococcus spp.	Classe Funcional IV (NYHA)	Sexo feminino	Complicações perianulares^‡^
Hipertensão sistêmica	Cirurgia urgente	PSAP > 55 mmHg	FEVE	EI de valva protética
DMID/DMNID	Sexo (feminino)		Número de valvas/ próteses tratadas	Choque séptico
Procedimento valvar múltiplo	EuroSCORE ≥ 10%		Microorganismo isolado na hemocultura	Trombocitopenia^§^
Inotrópicos ou BIA no pré-operatório			Presença de abscesso	Microrganismo virulento^//^
CRM prévia				
Cirurgia valvar prévia				
Insuficiência Renal (HD) ou Cr sérica > 2 mg/dL				
Urgência				

*Taquicardia ventricular sustentada, fibrilação ventricular, fibrilação atrial, flutter atrial ou bloqueio atrioventricular de terceiro grau. ^†^Abscessos ou outros achados ecocardiográficos sugestivos de infecção invasiva (comunicação entre câmaras, dissecção da parede ou extensa deiscência da válvula). ^‡^Abscesso, pseudoaneurisma, fístula ou deiscência protética. ^§^ < 150.000 plaquetas/mm^3^. //Staphylococcus aureus ou fungos. IMC: índice de massa corporal; CRM: cirurgia de revascularização miocárdica; CCS: Canadian Cardiovascular Society; DPOC: doença pulmonar obstrutiva crônica; Cr: creatinina; eTFG: estimativa da taxa de filtração glomerular; HD: hemodiálise; DMID: diabetes mellitus insulino-dependente; DMNID: Diabetes mellitus não insulino-dependente; EI: endocardite infecciosa; FEVE: fração de ejeção do ventrículo esquerdo; IAM: infarto agudo do miocárdio; NYHA: New York Heart Association; PSAP: pressão sistólica da artéria pulmonar.

A insuficiência renal aguda foi definida como qualquer um dos seguintes critérios: aumento ≥ a 0,3 mg/dL da creatinina em 48 horas ou aumento de ≥ 1,5 vezes da creatinina em relação ao valor basal, conhecido ou que se presume nos 7 dias anteriores; volume urinário < 0,5mL/kg/h em 6 horas.^15^ O estado crítico no pré-operatório foi definido como a presença de qualquer uma das caracteríticas a seguir durante a mesma internação hospitalar que a da cirurgia: taquicardia/fibrilação ventricular ou morte súbita recuperada; massagem cardíaca; ventilação ventilação mecânica antes da sala anestésica; administração de inotrópicos; uso de balão de contrapulsação/dispositivo de assistência ventricular antes da sala anestésica ou insuficiência renal aguda (anúria ou oligúria <10 mL/h).^9^ A EI ativa (ainda com tratamento antibiótico no momento da cirurgia), doença pulmonar crônica, arteriopatia extracardíaca, mobilidade reduzida (comprometimento grave de mobilidade secundária à disfunção neuromúsculo-esquelética), infarto do miocárdio recente (≤ 90 dias), hipertensão arterial pulmonar grave (pressão arterial pulmonar sistólica > 55mmHg), disfunção renal grave (ClCr < 50mL/min), e urgência de cirurgia também foram definidas conforme o EuroSCORE II.^9^

### Análise Estatística

Os dados foram coletados diretamente dos prontuários eletrônicos dos pacientes e analisados utilizando os *softwares* IBM SPSS 21.0, MedCalc 12.5 e OpenEpi 3.01.^16^ Os dados qualitativos foram exibidos por meio de frequência absoluta e relativa; média (desvio padrão) ou mediana (intervalo interquartil) foram utilizadas para as análises quantitativas. A normalidade da distribuição de cada variável foi avaliada por meio do teste Shapiro-Wilk. A calibração (expressa pela razão entre a mortalidade observada e a esperada [O/E], isto é, a razão de mortalidade padronizada [RMP]) e a capacidade discriminatória (expressa pela área sob a curva ROC [ASC]) dos escores foi avaliada. Para calcular a RPM com intervalo de confiança (IC) de 95%, utilizamos o teste exato de Mid-p modificado por Miettinen. A comparação da ASC foi realizada pelo teste de DeLong. Valores de p < 0,05 foram considerados estatisticamente significantes.

## Resultados

Durante o período estudado, foram incluídos 107 pacientes submetidos à cirurgia cardíaca na instituição durante a fase aguda da EI. A idade média dos pacientes foi de 58,1 ± 14,5 anos, sendo 24,3% mulheres. A EI aórtica isolada foi a forma predominante de EI (43,9%). As características dos pacientes e os detalhes cirúrgicos estão descritos na Tabela 2.

**Tabela 2:** – Características dos pacientes e variáveis cirúrgicas

VARIÁVEL	n = 107
Idade (anos)	58,1±14,5
Sexo feminino	26 (24,3)
Hipertensão arterial sistêmica	60 (56,1)
Classe funcional III/IV (NYHA)	53 (49,5)
Abscesso valvar	40 (37,4)
Cirurgia cardíaca prévia	35 (32,7)
Doença valvar degenerativa	31 (29,0)
HAP grave	31 (29,0)
Endocardite protética	31 (29,0)
Insuficiência renal aguda	30 (28,0)
Disfunção renal grave*	25 (26,0)
Diálise	22 (20,6)
Trombocitopenia	20 (18,7)
Estado crítico no pré-operatório	19 (17,8)
FEVE ≤ 50%	17 (15,9)
DMID	14 (13,1)
Endocardite infecciosa prévia	11 (10,3)
Valvulopatia reumática	10 (9,3)
Válvula aórtica bicúspide	8 (7,5)
Arteriopatia extracardíaca	8 (7,5)
IAM prévio	8 (7,5)
Doença pulmonar crônica	7 (6,5)
Mobilidade reduzida	7 (6,5)
IAM recente	3 (2,8)
Angina CCS classe 4	1 (0,9)
Localização da endocardite infecciosa	
Valva aórtica	47 (43,9)
Valva mitral	35 (32,7)
Valvas aórtica + mitral	20 (18,7)
Valva tricúspide	4 (3,7)
Valvas tricúspide + mitral	1 (0,9)
Microrganismo causador identificado	72 (67,3)
* Streptococcus viridans*	19 (17,8)
* Enterococcus sp.*	10 (9,3)
* Staphylococcus aureus*	9 (8,4)
Magnitude da intervenção	
Outra que não CRM isolada	81 (75,7)
Dois procedimentos	25 (23,4)
Três procedimentos	1 (0,9)
Urgência	
Urgente	98 (91,6)
Emergente	9 (8,4)
CRM associada	8 (7,5)
Tempo de circulação extracorpórea (min)	84,0 (65,0-110,0)
Tempo de isquemia (min)	65,0 (51,0-84,0)

CRM: cirurgia de revascularização do miocárdio; CCS: Canadian Cardiovascular Society; DMID: diabetes mellitus insulino-dependente; NYHA: New York Heart Association; HAP: hipertensão arterial pulmonar; FEVE: fração de ejeção do ventrículo esquerdo; IAM: infarto agudo do miocárdio. *Excluímos pacientes submetidos à hemodiálise no pré-operatório (n = 22; 20,6%) e aqueles cujos dados de peso corporal não estavam disponíveis (n = 11; 10,3%), o que tornou impossível calcular o clearance de creatinina. Dados expressos como média ± desvio padrão, n (%), ou mediana (intervalo interquartil).

O tamanho mediano das vegetações foi de 14,0 mm (9,25-18,0). Trinta e um pacientes (29,0%) apresentaram pelo menos 1 evento embólico, diagnosticado com base nos sintomas ou incidentalmente: 13 (12,1%) para o sistema nervoso central e 11 (10,3%) para o baço. Vinte e dois (20,6%) estavam em diálise no pré-operatório: 14 (13,1%) devido à doença renal crônica, 6 (5,6%) devido à insuficiência renal aguda, e 2 (1,9%) devido à doença renal crônica agudizada.

A cirurgia foi realizada com um tempo médio de 12,5 (6,0-22,25) dias do início da antibioticoterapia. A principal indicação para cirurgia foi insuficiência cardíaca (76,6%). O procedimento mais frequentemente realizado foi a troca valvar aórtica mecânica (n = 26; 24,3%), seguida da troca valvar aórtica biológica (n = 22; 20,6%) e da troca valvar mitral biológica (n = 22; 20,6%).

**Figura 1:**
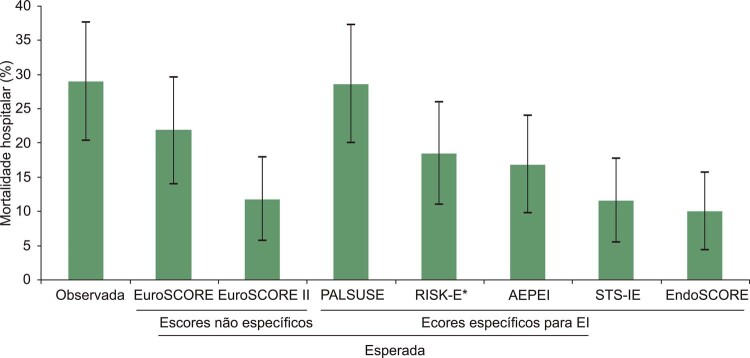
– Mortalidade hospitalar observada e esperada de acordo com os escores. *A mortalidade observada foi de 29,0%, exceto para o RISK-E, que foi de 28,4% (5 casos de endocardite infecciosa pulmonar/tricúspide foram excluídos, uma vez que eles não estão incluídos na análise desse escore). As barras de erro indicam intervalo de confiança de 95%.

A mortalidade hospitalar global foi de 29,0% (IC95%: 20,4-37,6%). Houve uma grande variação na mortalidade esperada dentre os escores, de 10,0% no EndoSCORE a 28,6% no escore PALSUSE (Figura 1). A melhor razão de mortalidade O/E foi obtida pelo escore PALSUSE (1,01; IC95%: 0,70-1,42; p=0,919), seguido pelo EuroSCORE logístico (1,3; IC95%: 0,92-1,87; p=0,123), conforme mostrado na Tabela 3. Todos os outros escores subestimaram significativamente a mortalidade hospitalar.

**Tabela 3:** – Razão de mortalidade observada/esperada e análise da curva ROC para os escores estudados

ESCORE	MORTALIDADE O/E *	IC95%	p	ASC	IC95%	p
**ESCORES NÃO ESPECÍFICOS PARA EI**						
EuroSCORE logístico	1,33	0,92-1,87	0,123	0,77	0,66-0,87	< 0,001
EuroSCORE II	2,46	1,70-3,45	< 0,001	0,69	0,58-0,80	0,002
**ESCORES ESPECÍFICOS PARA EI**						
STS-IE	2,50	1,73-3,50	< 0,001	0,67	0,56-0,79	0,005
PALSUSE	1,01	0,70-1,42	0,919	0,68	0,57-0,79	0,003
RISK-E	1,53	1,05-2,18	0,029	0,71	0,60-0,81	0,001
AEPEI	1,71	1,18-2,40	0,006	0,65	0,53-0,77	0,017
EndoSCORE	2,90	2,00-4,06	< 0,001	0,76	0,66-0,86	< 0,001

*A mortalidade observada foi de 29,0%, exceto para o RISK-E, que foi de 28,4% (5 casos de endocardite infecciosa pulmonar/tricúspide foram excluídos, uma vez que eles não estão incluídos na análise desse escore). O/E: observada/esperada; ASC: área sob a curva; IC: intervalo de confiança; EI: endocardite infecciosa.

O EuroSCORE logístico teve o maior poder discriminatório (ASC: 0,77), como pode ser visto na Tabela 3, sendo significativamente superior ao do EuroSCORE II (p = 0,03), STS-IE (p = 0,03), PALSUSE (p = 0,03), AEPEI (p = 0,03), e RISK-E (p = 0,02), embora não significativamente quando comparado com o EndoSCORE (p = 0,90). Todas as outras comparações foram não significativas, exceto EndoSCORE vs. AEPEI (p = 0,03).

## Discussão

Nesta coorte de pacientes submetidos à cirurgia cardíaca devido a EI, a melhor razão de mortalidade O/E e poder discriminatório foram obtidos pelo escore PALSUSE (1,01) e pelo EuroSCORE (ASC: 0,77), respectivamente. O EuroSCORE logístico, que apresentou a segunda melhor razão O/E (1,3), apresentou poder discriminatório significativamente melhor do que o escore PALSUSE (ASC: 0,68; p=0,03).

A ASC, também conhecida como estatística C ou índice C, é um marcador de precisão diagnóstica global^17^ e uma medida efetiva e combinada de sensibilidade e especificidade.^18^ O poder discriminatório é considerado excelente se > 0,80, muito bom se > 0,75, e bom (aceitável) se > 0,70. Além disso, avaliamos a calibração utilizando a razão de mortalidade O/E. Idealmente, essa razão é 1, isto é, a mortalidade observada é igual à mortalidade esperada, indicando um modelo preditivo perfeitamente calibrado. Um valor de O/E > 1 significa que o modelo subestimou a mortalidade, ao passo que um valor < 1 significa que o modelo superestimou a mortalidade. Se o IC de 95% para a razão de mortalidade O/E cruza 1, o modelo está bem calibrado.^19^ Contudo, é possível que um modelo de risco tenha boa calibração, mas pouca discriminação, e vice-versa. A discriminição é mais importante que a calibração; um modelo pode ser recalibrado ou reajustado conforme a prática for aprimorada, porém se o modelo for construído levando em conta fatores de risco incorretos, sua discriminação não poderá ser melhorada.^20^ Embora o EndoSCORE não tenha apresentado um poder discriminatório significativamente pior quando comparado com o EuroSCORE logístico, ele subestimou significativamente a mortalidade hospitalar; desse modo, a realização de ajustes seria necessária. Na nossa coorte, o EuroSCORE logístico demonstrou ser o melhor preditor de risco de mortalidade.

O microrganismo causador foi identificado em apenas 67,3% dos casos nesta coorte, diferentemente das coortes de validação dos escores específicos para EI, nas quais a taxa de detecção foi de 81,0-86,6%.^10-12^ De modo semelhante, o *Staphylococcus aureus*, que causa uma infecção agressiva e, muitas vezes, fatal,^21^ foi o microrganismo causador em apenas 8,4% dos casos, ao passo que, nas coortes de validação, esse percentual variou de 17,5 a 19,9%.^11,12^ Esses dois fatores provavelmente explicam, ao menos em parte, a baixa precisão dos escores específicos para EI na nossa coorte. O mesmo ocorreu com outros itens incluídos nos escores específicos, tais como a classe funcional IV da NYHA no escore AEPEI^10^(37,7 *vs.* 20,6%), FEVE ≤ 50% no EndoSCORE^7^ (35,9 *vs. *15,9%), choque cardiogênico e trombocitopenia no RISK-E^12^(17,9 e 29,2% no estudo original vs. 11,2 e 18,7% neste estudo, respectivamente); embora fortemente associados com a mortalidade, esses fatores não foram significativamente predominantes na nossa coorte.

O EuroSCORE II, o mais comumente utilizado para avaliação do risco pré-operatório na prática clínica atual, subestimou em 2,5 vezes a mortalidade observada e teve baixo poder discriminatório (ASC: 0,69). A coorte original do estudo EuroSCORE II tinha uma porcentagem muito baixa de pacientes com com EI ativa (2,2%);^9^ consequentemente, é difícil generalizar os resultados do EuroSCORE II para as populações com EI. Em uma análise de 149 pacientes submetidos à cirurgia cardíaca em razão de EI ativa em dois centros de referência franceses, Patrat-Delon et al.,^6^ observaram que, apesar de o EuroSCORE II ter bom poder discriminatório (ASC: 0,78; IC95%: 0,70-0,84), seus resultados deveriam ser interpretados com cautela durante a fase aguda da EI, porque esse escore subestimou a mortalidade pós-operatória em 5-10% na metade dos pacientes com mortalidade prevista de >10%. No Brasil, Oliveira et al.,^22^ conduziram o único outro estudo até hoje que avaliou o uso de predição em pacientes com EI ativa submetidos à cirurgia cardíaca. Nesse estudo, que incluiu 88 pacientes, o EuroSCORE II subestimou significativamente a mortalidade hospitalar, com uma razão de mortalidade O/E de 2,31 (IC95%: 1,41-3,58; p = 0,002). A análise da curva ROC não foi realizada.

Os pacientes com EI ativa já haviam sido sub-representados na coorte do EuroSCORE,^8^ na qual a EI ativa estava presente em apenas 3,6% de todos os pacientes submetidos à cirurgia valvar. Madeira et al.,^23^ em um estudo com 128 pacientes submetidos à cirurgia cardíaca devido a EI ativa, compararam o EuroSCORE e o EuroSCORE II para predição de mortalidade perioperatória. Eles observaram que o padrão de calibração diferiu entre os dois escores: o EuroSCORE mostrou uma tendência progressiva superestimar, ao passo que o EuroSCORE II tendeu a subestimar a mortalidade. Por outro lado, assim como no presente estudo, Mestres et al.,^24^ em um estudo com 181 pacientes com EI (93,2% ativa), descreveram bom poder discriminatório (ASC: 0,84) e uma mortalidade esperada (27,1%) muito semelhante àquela da observada (28,8%; razão O/E: 1,1).

A necessidade de uma ferramenta de estratificação específica, útil tanto para a informação pré-operatória ao paciente quanto para a tomada de decisão à beira do leito, surge das peculiaridades da cirurgia da EI em relação à cirurgia cardíaca em geral: os defechos pós-operatórios podem ser influenciados não somente por questões anatômicas ou funcionais cardiovasculares, mas também por fatores microbiológicos e infecciosos sistêmicos.^25^Mais recentemente, foram desenvolvidos novos escores de risco específicos para a EI. Eles incorporam alguns fatores específicos da EI (tais como culturas microbiológicas, formação de abscesso e sepse), que são conhecidos preditores independentes de mortalidade. Os escores específicos para EI vêm demonstrando maior precisão na predição de mortalidade do que os escores de risco tradicionais.^26^

Dentre os escores específicos para EI analisados, apenas o PALSUSE^10^ e o RISK-E^12^ tinham coortes de derivação limitadas a pacientes com EI ativa. O escore PALSUSE,^10^ que incorpora o EuroSCORE na sua composição, foi derivado de um estudo de coorte prospectiva com 437 pacientes que foram submetidos à cirurgia na na fase aguda da EI. Os dados foram coletados em 26 hospitais espanhóis. A mortalidade hospitalar foi de 24,3%, variando de 0% em pacientes com um escore de 0 a 45,4% naqueles com um escore ≥ 4. A ASC foi de 0,84 (IC95%: 0,79-0,88), indicando capacidade discriminatória satisfatória. O RISK-E^12^ foi desenvolvido a partir de uma pesquisa realizada em três centros de saúde terciários da Espanha, que buscavam prever a mortalidade hospitalar em 424 pacientes com EI ativa aórtica/mitral submetidos à cirurgia cardíaca. A ASC foi de 0,82 (IC95%: 0,75-0,88). A probabilidade preditiva da mortalidade pós-operatória variou de 3% para um paciente com um escore de 0 a 97% para um paciente com o escore mais alto possível de 68. Uma comparação das ASCs apresentou desempenho preditivo superior estatisticamente significativo do RISK-E (p = 0,01), em relação ao EuroSCORE, EuroSCORE II, ou PALSUSE.

De 2000 a 2015, dados de 2.715 pacientes com endocardite (70,1% ativa) que foram submetidos à cirurgia em 26 centros cirúrgicos cardíacos italianos foram coletados retrospectivamente. Esse amplo estudo^7^ forneceu um modelo de risco logístico para prever a mortalidade precoce (dentro de 30 dias da cirurgia): o EndoSCORE. A ASC foi de 0,84 (IC95%: 0,81-0,86). No nosso estudo, esse escore foi testado para prever o risco de morte durante a internação, independentemente do tempo de permanência hospitalar, e 5 das 31 mortes (16,1%) ocorreram 30 dias após a cirurgia (mortalidade precoce: 24,3%). Essa diferença pareceu ter pouco efeito sobre o desempenho do escore, que igualmente subestimou a mortalidade precoce (razão O/E: 2,4; ASC: 0,77 [IC95%: 0,66-0,88]).

Embora o AEPEI^11^ seja um escore específico para EI, ele não inclui variáveis específicas de EI no seu modelo final. Ele foi desenvolvido a partir de um estudo prospectivo com 361 pacientes consecutivos que haviam sido submetidos à cirurgia em razão de EI (76,2% ativa) em oito centros cirúrgicos cardíacos europeus. Cinquenta e seis pacientes (15,5%) morreram após a cirurgia, e a ASC foi de 0,78 (IC95%: 0,73-0,82). Na população do estudo, o escore AEPEI teve poder discriminatório equivalente àquele do EuroSCORE II (p = 0,4) e mostrou superioridade em relação ao EuroSCORE logístico (p = 0,0026) e o escore PALSUSE (p = 0,047).

Assim como o escore AEPEI, o STS-IE^2^ não inclui variávies específicas para EI. Ele foi desenvolvido a partir do banco de dados de cirurgia cardíaca adulta da *Society of Thoracic Surgeon*s (STS), que foi estabelecido em 1989, incluindo dados de 3 milhões de procedimentos cardíacos de mais de 90% dos centros cirúrgicos cardíacos da América do Norte. De 2002 a 2008, 19.543 cirurgias foram realizadas em pacientes com EI (51,5% ativa), com uma mortalidade de 8,2%. O STS-IE demonstrou boa capacidade preditiva, com uma ASC de 0,76.

Algumas limitações do nosso estudo devem ser mencionadas. Em primeiro lugar, o desenho retrospectivo pode ter influenciado a qualidade e a consistência dos dados coletados. O tamanho da amostra relativamente pequeno também é uma fonte de preocupação. Finalmente, o fato de o estudo ter sido conduzido em um único centro pode limitar a validade externa dos nossos achados.

## Conclusões

Nossos resultados mostraram que, apesar da disponibilidade de escores recentes específicos para EI, e considerando-se o equilíbrio entre os índices, o EuroSCORE logístico pareceu ser o melhor preditor de risco de mortalidade na nossa coorte de pacientes com EI, admitidos durante um período de 10 anos, levando-se em conta a calibração (razão O/E: 1,3) e a capacidade discriminante (ASC: 0,77). Esse achado tem implicações clínicas, já que o EuroSCORE II é o escore mais frequentemente utilizado na avaliação pré-operatória. Faz-se necessária a validação local de escores específicos para avaliar o risco pré-operatório dos pacientes com EI.
