# Supplementary material for: Análise de Escores de Risco para Predição de Mortalidade em Pacientes Submetidos à Cirurgia Cardíaca por Endocardite
Source: Arq Bras Cardiol. 2020 Apr 6;114(3):518–24. [Article in Portuguese] doi: 10.36660/abc.20190050 (PMC7792725; doi:10.36660/abc.20190050)
Supplement: Translation

# Translation: Analysis of Risk Scores to Predict Mortality in Patients Undergoing Cardiac Surgery for Endocarditis

**Authors:** Fernando Pivatto, Clarissa Carmona de Azevedo Bellagamba, Eduardo Gatti Pianca, Fernando Schmidt Fernandes, Maurício Butzke, Stefano Boemler Busato, Miguel Gus

**Affiliations:** 1 BrazilHospital de Clínicas de Porto Alegre, Porto Alegre, RS – Brazil

**Keywords:** Cardiovascular Surgical Procedures/mortality, Endocarditis/complications, Hospital Mortality, Risk Assessment

## Abstract

**Background:**

Risk scores are available for use in daily clinical practice, but knowing which one to choose is still fraught with uncertainty.

**Objectives:**

To assess the logistic EuroSCORE, EuroSCORE II, and the infective endocarditis (IE)-specific scores STS-IE, PALSUSE, AEPEI, EndoSCORE and RISK-E, as predictors of hospital mortality in patients undergoing cardiac surgery for active IE at a tertiary teaching hospital in Southern Brazil.

**Methods:**

Retrospective cohort study including all patients aged ≥ 18 years who underwent cardiac surgery for active IE at the study facility from 2007-2016. The scores were assessed by calibration evaluation (observed/expected [O/E] mortality ratio) and discrimination (area under the ROC curve [AUC]). Comparison of AUC was performed by the DeLong test. A p < 0.05 was considered statistically significant.

**Results:**

A total of 107 patients were included. Overall hospital mortality was 29.0% (95%CI: 20.4-37.6%). The best O/E mortality ratio was achieved by the PALSUSE score (1.01, 95%CI: 0.70-1.42), followed by the logistic EuroSCORE (1.3, 95%CI: 0.92-1.87). The logistic EuroSCORE had the highest discriminatory power (AUC 0.77), which was significantly superior to EuroSCORE II (p = 0.03), STS-IE (p = 0.03), PALSUSE (p = 0.03), AEPEI (p = 0.03), and RISK-E (p = 0.02).

**Conclusions:**

Despite the availability of recent IE-specific scores, and considering the trade-off between the indexes, the logistic EuroSCORE seemed to be the best predictor of mortality risk in our cohort, taking calibration (O/E mortality ratio: 1.3) and discrimination (AUC 0.77) into account. Local validation of IE-specific scores is needed to better assess preoperative surgical risk. (Arq Bras Cardiol. 2020; 114(3):518-524)

## Introduction

Despite advances in medical and surgical treatment, infective endocarditis (IE) is associated with substantial morbidity and risk of death.^1^ Surgical correction of active IE is associated with the highest mortality of any valve disease, with overall rates of in-hospital mortality exceeding 20%.^2^

Surgery is currently performed in 50 to 60% of patients with IE.^3^ The indications are: heart failure (usually related to valve dysfunction), uncontrolled infection (often associated with perivalvular extension and atrioventricular conduction defects), and prevention of systemic embolism.^4^ Although these indications are clear, their practical application relies largely on the patient’s clinical status, comorbidities and operative risk.^5^

Risk prediction models for cardiac surgery have been developed to provide information on risks for both clinicians and patients and to guide decision-making.^6^ Assessment of surgical risk helps to measure the quality of healthcare service, and risk profile is essential to differentiate patients by severity of health status. Likewise, being aware of a patient’s risk can allow implementation of individualized strategies to prevent complications.^7^ Although risk scores are available for use in daily clinical practice, knowing which one to choose is still fraught with uncertainty. Within this context, the aim of the present study was to assess the logistic EuroSCORE,^8^ EuroSCORE II,^9^ and the IE-specific scores STS-IE,^2^ PALSUSE,^10^AEPEI,^11^ EndoSCORE^7^ and RISK-E,^12^ as predictors of hospital mortality in patients undergoing cardiac surgery for active IE at a tertiary teaching hospital in Southern Brazil.

## Methods

This retrospective cohort study included all patients aged ≥ 18 years who underwent cardiac surgery for active IE at Hospital de Clínicas de Porto Alegre (HCPA), a tertiary teaching hospital in Southern Brazil, from 2007 to 2016. Only patients with definite IE based on the modified Duke criteria^13^ were enrolled. Patients were identified through surgical schedules and a keyword search of the HCPA electronic medical records system. The present study was approved by the HCPA Research Ethics Committee (protocol 16-0632).

Preoperative risk of death was estimated through the mean logistic EuroSCORE^8^ and EuroSCORE II^9^, in addition to the IE-specific scores STS-IE,^2^ PALSUSE,^10^ AEPEI,^11^ EndoSCORE^7^ and RISK-E^12^ (Table 1). Death during hospitalization, regardless of length of stay, was defined as hospital mortality. Creatinine clearance (CC) was estimated through the Cockcroft-Gault formula.^14^

**Table 1:** – Infective endocarditis-specific scores analyzed in the present study

NON-SPECIFIC SCORES				
EuroSCORE, 1999^8^			EuroSCORE II, 2012^9^	
Active endocarditis			Active endocarditis	
Age			Age	
Critical preoperative state			CCS class 4 angina	
Cr > 200 µmol/L			Chronic pulmonary dysfunction	
Extracardiac arteriopathy			Critical preoperative state	
Female sex			Extracardiac arteriopathy	
LVEF			Female sex	
Neurological dysfunction			IDDM	
Non-coronary surgery			LVEF	
Pulmonary disease			NYHA class	
Previous cardiac surgery			Poor mobility	
Recent MI			Previous cardiac surgery	
sPAP > 60 mmHg			Recent MI	
Thoracic aortic surgery			Renal dysfunction	
Unstable angina			sPAP	
Urgency			Thoracic aortic surgery	
Ventricular septal rupture			Urgency	
			Weight of procedure	
**IE-SPECIFIC SCORES**				
**STS-IE, 2011**^**2**^	**PALSUSE, 2014**^**10**^	**AEPEI, 2017**^**11**^	**EndoSCORE, 2017**^**12**^	**RISK-E, 2017**^**13**^
Active endocarditis	Prosthetic valve IE	BMI > 27Kg/m^2^	Age	Acute renal insufficiency
Arrhythmia*	Age	Critical preoperative state	COPD	Age
Cardiogenic shock	Large intracardiac destruction†	eGFR < 50mL/min	Cr ≥ 2mg/dL	Cardiogenic shock
Chronic lung disease	Staphylococcus spp.	NYHA class IV	Female sex	Periannular complications^‡^
Systemic hypertension	Urgent surgery	sPAP > 55 mmHg	LVEF	Prosthetic-valve IE
IDDM/NIDDM	Sex (female)		Number of treated valves/prostheses	Septic shock
Multiple valve procedure	EuroSCORE ≥ 10%		Pathogen isolated on blood specimen culture	Thrombocytopenia^§^
Preoperative IABP or inotropes			Presence of abscess	Virulent microorganism^//^
Prior CABG				
Prior valve surgery				
Renal failure (HD) or Cr > 2 mg/dL				
Urgency				

*Sustained ventricular tachycardia, ventricular fibrillation, atrial fibrillation, atrial flutter or third degree heart block. ^†^Abscesses or other echocardiography findings suggested the infection was invasive (communication between chambers, wall dissection or large valvular dehiscence). ^‡^Abscess, pseudoaneurysm, fistula or prosthetic dehiscence. ^§^ < 150,000 platelets/mm^3^. ^//^Staphylococcus aureus or fungi. BMI: body mass index; CABG: coronary artery bypass graft; CCS: Canadian Cardiovascular Society; COPD: chronic obstructive pulmonary disease; Cr: creatinine; eGFR: estimated glomerular filtration rate; HD: hemodialysis; IDDM: insulin-dependent diabetes mellitus; NIDDM: non-insulin-dependent diabetes mellitus; IE: infective endocarditis; LVEF: left ventricular ejection fraction; MI: myocardial infarction; NYHA: New York Heart Association; sPAP: systolic pulmonary artery pressure.

Acute renal insufficiency was defined as any of the following: increase in creatinine by ≥ 0.3 mg/dL within 48 hours or to ≥ 1.5 times baseline, which is known or presumed to have occurred within the past 7 days; or urinary output < 0.5 mL/kg/h for 6 hours.^15^ Critical preoperative state was defined if any one or more of the following occurred preoperatively during the same hospital admission as the operation: ventricular tachycardia/fibrillation or aborted sudden death; cardiac massage; ventilation before arrival at the anesthesia suite; administration of inotropes; intra-aortic balloon counterpulsation/ventricular-assist device placement before arrival at the anesthesia suite or acute renal failure (anuria or oliguria < 10 mL/h).^9^ Active IE (still on antibiotic treatment for IE at time of surgery), chronic pulmonary disease, extracardiac arteriopathy, poor mobility (severe impairment of mobility secondary to musculoskeletal or neurological dysfunction), recent myocardial infarction (≤ 90 days), severe pulmonary arterial hypertension (systolic pulmonary artery pressure > 55mmHg), severe renal dysfunction (CC < 50mL/min) and urgency of surgery were also defined as in the EuroSCORE II study.^9^

### Statistical Analysis

Data were collected directly from the patients’ electronic charts and analyzed in IBM SPSS Statistics for Windows, version 21.0; MedCalc, version 12.5; and OpenEpi, version 3.01.^16^ Qualitative data were reported as absolute and relative frequency; mean (standard deviation) or median (interquartile range) were used for quantitative variables. The normality of distribution of each variable was evaluated using the Shapiro-Wilk test. Calibration (expressed by the observed/expected [O/E] mortality ratio, i.e., the standard mortality ratio [SMR]) and discriminant ability (by area under the ROC curve [AUC]) of the scores were evaluated. To calculate the SMR with a 95% confidence interval (CI), we used the mid-P exact test with Miettinen’s modification. Comparison of AUC was performed by the DeLong test. P-values < 0.05 were considered statistically significant.

## Results

During the study period, 107 patients underwent cardiac surgery at the study facility while in the acute phase of IE and were included. Mean age was 58.1 ± 14.5 years and 24.3% were female. Isolated aortic IE was the most prevalent form of IE (43.9%). Patient characteristics and surgical details are described in Table 2.

**Table 2:** – Patient characteristics and surgical details.

VARIABLE	n = 107
Age (years)	58.1±14.5
Female sex	26 (24.3)
Hypertension	60 (56.1)
NYHA III/IV	53 (49.5)
Abscess	40 (37.4)
Previous cardiac surgery	35 (32.7)
Degenerative valve disease	31 (29.0)
Severe PAH	31 (29.0)
Prosthetic endocarditis	31 (29.0)
Acute renal insufficiency	30 (28.0)
Severe renal dysfunction*	25 (26.0)
Dialysis	22 (20.6)
Thrombocytopenia	20 (18.7)
Critical preoperative state	19 (17.8)
LVEF ≤ 50%	17 (15.9)
IDDM	14 (13.1)
Previous infective endocarditis	11 (10.3)
Rheumatic valvulopathy	10 (9.3)
Bicuspid aortic valve	8 (7.5)
Extracardiac arteriopathy	8 (7.5)
Previous MI	8 (7.5)
Chronic lung disease	7 (6.5)
Poor mobility	7 (6.5)
Recent MI	3 (2.8)
CCS class 4 angina	1 (0.9)
Location of infective endocarditis	
Aortic	47 (43.9)
Mitral	35 (32.7)
Aortic + Mitral	20 (18.7)
Tricuspid	4 (3.7)
Tricuspid + Mitral	1 (0.9)
Identified causative microorganism	72 (67.3)
* Streptococcus viridans*	19 (17.8)
* Enterococcus sp.*	10 (9.3)
* Staphylococcus aureus*	9 (8.4)
Magnitude of intervention	
Single, non-CABG	81 (75.7)
Two procedures	25 (23.4)
Three procedures	1 (0.9)
Urgency	
Urgent	98 (91.6)
Emergent	9 (8.4)
Associated CABG	8 (7.5)
Extracorporeal circulation time (min)	84.0 (65.0-110.0)
Cross-clamp time (min)	65.0 (51.0-84.0)

CABG: coronary artery bypass graft; CCS: Canadian Cardiovascular Society; IDDM: insulin-dependent diabetes mellitus; NYHA: New York Heart Association; PAH: pulmonary arterial hypertension; LVEF: left ventricular ejection fraction; MI: myocardial infarction. *We excluded patients on preoperative hemodialysis (n = 22; 20.6%) and those for whom body weight data were unavailable (n = 11; 10.3%), which makes it impossible to calculate the creatinine clearance. Data expressed as mean ± standard deviation, n (%), or median (interquartile range).

The median vegetation size was 14.0 (9.25-18.0) millimeters. Thirty-one patients (29.0%) experienced at least one embolic event, diagnosed on the basis of symptoms or by incidental detection: 13 (12.1%) to the central nervous system and 11 (10.3%) to the spleen. Twenty-two (20.6%) were on preoperative dialysis: 14 (13.1%) due to chronic kidney disease, 6 (5.6%) due to acute renal failure, and 2 (1.9%) due to acute-on-chronic renal failure.

Surgery was performed with a median delay of 12.5 (6.0-22.25) days start of antibiotic therapy. The leading indication for surgery was heart failure (76.6%). The most frequently performed procedure was mechanical aortic valve replacement (n = 26, 24.3%), followed by bioprosthetic aortic valve replacement (n = 22, 20.6%) and bioprosthetic mitral valve replacement (n = 22, 20.6%).

Overall hospital mortality was 29.0% (95%CI: 20.4-37.6%). There was a wide variation in expected mortality among the scores, ranging from 10.0% in EndoSCORE to 28.6% in PALSUSE score (Figure 1). The best O/E mortality ratio was achieved by the PALSUSE score (1.01, 95%CI: 0.70-1.42; p = 0.919), followed by the EuroSCORE (1.3, 95%CI: 0.92-1.87; p = 0.123), as seen in Table 3. All other scores significantly underestimated hospital mortality.

**Figure 1:** – Observed and expected hospital mortality according scores. *Observed mortality was 29.0%, except for the RISK-E score, which was 28.4% (5 right-sided infective endocarditis cases were excluded, since they are not included in this score analysis). Error bars represent 95% confidence intervals.

**Table 3:** – Observed/expected mortality ratio and ROC curve analysis for the studied scores

SCORE	O/E MORTALITY*	95%CI	p	AUC	95%CI	p
**NON-SPECIFIC SCORES**						
Logistic EuroSCORE	1.33	0.92-1.87	0.123	0.77	0.66-0.87	<0.001
EuroSCORE II	2.46	1.70-3.45	<0.001	0.69	0.58-0.80	0.002
**IE-SPECIFIC SCORES**						
STS-IE	2.50	1.73-3.50	<0.001	0.67	0.56-0.79	0.005
PALSUSE	1.01	0.70-1.42	0.919	0.68	0.57-0.79	0.003
RISK-E	1.53	1.05-2.18	0.029	0.71	0.60-0.81	0.001
AEPEI	1.71	1.18-2.40	0.006	0.65	0.53-0.77	0.017
EndoSCORE	2.90	2.00-4.06	<0.001	0.76	0.66-0.86	<0.001

*Observed mortality was 29.0%, except for the RISK-E score, which was 28.4% (5 right-sided infective endocarditis cases were excluded, since they are not included in this score analysis). O/E: observed/expected; AUC: area under the curve; CI: confidence interval; IE: infective endocarditis.

The logistic EuroSCORE had the highest discriminatory power (AUC 0.77), as seen in Table 3, which was significantly superior to that of EuroSCORE II (p = 0.03), STS-IE (p = 0.03), PALSUSE (p = 0.03), AEPEI (p = 0.03), and RISK-E (p = 0.02), and non-significantly so when compared to EndoSCORE (p = 0.90). All other comparisons were non-significant, except for EndoSCORE versus AEPEI score (p = 0.03).

## Discussion

In this cohort of patients undergoing cardiac surgery for active IE, the best O/E mortality ratio and discriminatory power were achieved by the PALSUSE score (1.01) and the logistic EuroSCORE (AUC 0.77), respectively. The logistic EuroSCORE, which had the second best O/E ratio (1.3), also had significantly better discriminatory power than PALSUSE (AUC 0.68; p = 0.03).

AUC, also known as the c-statistic or c-index, is a marker of overall diagnostic accuracy^17^ and an effective and combined measure of sensitivity and specificity.^18^ Discriminative power is thought to be excellent if the AUC is > 0.80, very good if > 0.75, and good (acceptable) if > 0.70. We also evaluated calibration using the O/E mortality ratio. Ideally, this ratio will be 1, i.e., the observed mortality equals expected mortality, denoting a perfectly calibrated predictive model. An O/E value > 1 means the model underestimates mortality, while a value < 1 means the model overestimates mortality. If the 95%CI of the O/E mortality ratio crosses 1, the model is well calibrated.^19^ Nevertheless, it is possible for a risk model to have good calibration but poor discrimination, and vice versa. Discrimination is more important than calibration; a model can be recalibrated or adjusted as practice improves, but if the model is built on the wrong risk factors, its discrimination cannot be improved.^20^ Although the EndoSCORE did not show significantly worse discriminative power than the logistic EuroSCORE, it did significantly underestimate hospital mortality; thus, adjustments are required. In our cohort, the logistic EuroSCORE seemed to be the best predictor of mortality risk.

The causative microorganism was identified in only 67.3% of cases in this cohort, unlike in the validation cohorts of the IE-specific scores, in which the detection rate was 81.0-86.6%.^10-12^ Similarly, *Staphylococcus aureus*, which causes an aggressive and often fatal infection,^21^ was the causative microorganism in only 8.4% of cases, while in the validation cohorts this percentage ranged from 17.5 to 19.9%.^11,12^These two factors probably explain, at least partly, the low accuracy of IE-specific scores in our cohort. The same occurred with other items included in specific scores, such as NYHA class IV in the AEPEI score^10^ (37.7 vs. 20.6%), LVEF ≤ 50% in the EndoSCORE^7^ (35.9 vs. 15.9%), cardiogenic shock and thrombocytopenia in the RISK-E score^12^ (17.9 and 29.2% in the original study vs. 11.2 and 18.7% in the present study, respectively); although strongly associated with mortality, these factors were not significantly prevalent in our cohort.

EuroSCORE II, the most commonly used score for preoperative risk assessment in current clinical practice, underestimated the observed mortality 2.5-fold and had poor discriminatory power (AUC = 0.69). The original EuroSCORE II study cohort had a very low percentage of patients with active IE (2.2%);^9^ therefore, it is difficult to generalize EuroSCORE II results for IE populations. In an analysis of 149 patients undergoing cardiac surgery for active IE at two French referral centers for cardiac surgery, Patrat-Delon et al.^6^observed that, although EuroSCORE II showed good power of discrimination (AUC = 0.78; 95%CI: 0.70-0.84), its results should be interpreted with caution during the acute phase of IE, because it also underestimated postoperative mortality by 5-10% in half of patients with predicted mortality >10%. In Brazil, Oliveira et al.^22^ conducted the only other study to date to evaluate a prediction score in patients with active IE undergoing heart surgery. In this study, which included 88 patients, the EuroSCORE II significantly underestimated hospital mortality, with a mortality ratio O/E of 2.31 (95%CI: 1.41-3.58; p = 0.002). ROC curve analysis was not performed.

Patients with active IE were already underrepresented in the EuroSCORE cohort,^8^ in which active IE was present in only 3.6% of all valve surgery patients. Madeira et al.^23^ in a study including 128 patients who underwent heart surgery for active IE, compared EuroSCORE and EuroSCORE II for perioperative mortality prediction. They observed that the pattern of calibration differed between the scores: EuroSCORE showed a progressive trend towards overprediction, whereas EuroSCORE II tended to underpredict mortality. On the other hand, as in the present study, Mestres et al.^24^ in a study including 181 patients with IE (93.2% active), described good discriminatory power (AUC 0.84) and an expected mortality (27.1%) very similar to that observed (28.8%; O/E ratio: 1.1).

The need for a dedicated stratification tool, useful both for preoperative patient information and for bedside decision-making, arises from the peculiarities of IE surgery compared with general cardiac surgery: postoperative outcomes may be influenced not only by cardiovascular anatomic and functional issues, but also by systemic infective and microbiological factors.^25^ More recently, new IE-specific risk scores have been developed. They incorporate some IE-specific factors (such as microbiological cultures, abscess formation and sepsis) that are known to be independent predictors of mortality. IE-specific scores have demonstrated greater accuracy for mortality prediction than classical risk scores.^26^

Among the IE-specific scores analyzed, only the PALSUSE^10^and RISK-E^12^ scores had derivation cohorts limited to patients with active IE. The PALSUSE score,^10^ which incorporates the EuroSCORE in its composition, was derived from a prospective cohort study including 437 patients who underwent surgery in the acute phase of IE. Data were collected in 26 Spanish hospitals. In-hospital mortality was 24.3%, ranging from 0% in patients with a score of 0 to 45.4% in those with a score ≥4. AUC was 0.84 (95%CI: 0.79-0.88), indicating satisfactory discriminatory ability. The RISK-E score^12^ was developed from research performed in three tertiary care centers in Spain, which sought to predict in-hospital mortality in 424 patients with active left-sided IE undergoing cardiac surgery. AUC was 0.82 (95%CI: 0.75-0.88). The predicted probability of postoperative mortality ranged from 3% for a patient with a score of 0 to 97% for a patient with the highest possible score of 68. A comparison of AUCs showed a statistically significant superior predictive performance of the RISK-E score (p = 0.01) when compared with EuroSCORE, EuroSCORE II, or PALSUSE.

From 2000 to 2015, data from 2,715 patients with endocarditis (70.1% active) who underwent surgery at 26 Italian cardiac surgery centers were collected retrospectively. This large study^7^ provided a logistic risk model to predict early mortality (within 30 days of surgery): the EndoSCORE. AUC was 0.84 (95%CI: 0.81-0.86). In our study, this score was tested to predict death during hospitalization, regardless of length of stay, and 5 of 31 deaths (16.1%) occurred beyond 30 days after surgery (early mortality: 24.3%). This difference seemed to have little effect on the performance of the score, which also underestimated early mortality (O/E ratio: 2.4; AUC: 0.77 [95%CI: 0.66-0.88]).

The AEPEI score,^11^ despite being IE-specific, does not include IE-specific variables in its final model. It was developed in a prospective study including 361 consecutive patients who had undergone surgery for IE (76.2% active) at eight European cardiac surgery centers. Fifty-six patients (15.5%) died after surgery, and the AUC was 0.78 (95%CI: 0.73-0.82). In the study population, the AEPEI score had equivalent discriminatory power to that of the EuroSCORE II (p = 0.4) and was found to be better than the logistic EuroSCORE (p = 0.0026) and PALSUSE (p = 0.047).

Similarly to the AEPEI score, the STS-IE score^2^ does not include IE-specific variables. It was developed from the Society of Thoracic Surgeons (STS) adult cardiac surgery database, which was established in 1989, including data from nearly 3 million cardiac procedures from over 90% of cardiac surgical centers in North America. From 2002 through 2008, 19,543 operations were performed for IE (51.5% active), with a mortality of 8.2%. The STS-IE score demonstrated good predictive ability, with an AUC of 0.76.

Some limitations of our study should be mentioned. First, the retrospective design may have influenced the quality and consistency of the data collected. The relatively small sample size is also a source of concern. Finally, the fact that the study was conducted at a single center can limit the external validity of our findings.

## Conclusions

Our results showed that, despite the availability of recent IE-specific scores and considering the trade-off between the indexes, the logistic EuroSCORE seemed to be the best predictor of mortality risk in our 10-year IE cohort, considering calibration (O/E ratio: 1.3) and discriminant ability (AUC 0.77). This finding has clinical implications, as the EuroSCORE II is the score most commonly used score in preoperative evaluation. Local validation of the new IE-specific scores for preoperative risk assessment in this specific group of patients is needed.
